# Acid-Assisted Ball Mill Synthesis of Carboxyl-Functional-Group-Modified Prussian Blue as Sodium-Ion Battery Cathode

**DOI:** 10.3390/nano12081290

**Published:** 2022-04-11

**Authors:** Yu Luo, Jiayu Peng, Shengming Yin, Lihong Xue, Youwei Yan

**Affiliations:** State Key Laboratory of Material Processing and Die & Mould Technology, School of Materials Science and Engineering, Huazhong University of Science and Technology, Wuhan 430074, China; luoyu@hust.edu.cn (Y.L.); d201980327@hust.edu.cn (J.P.)

**Keywords:** Prussian blue, ball milling, surface modification, carboxyl functional group

## Abstract

Prussian blue attracts the attention of many researchers as a promising candidate for use in sodium-ion battery cathodes due to its open frameworks and high working potential. However, the interstitial water in its crystal structure and its poor electronic conductivity limits its performance in practical sodium-ion batteries. Here, acid-assisted ball milling synthesis was employed as a versatile method for the production of surface-modified Prussian blue. With (CH_3_COO)_2_Fe being used as the raw material, the Prussian blue produced using ball milling synthesis was modified by the carboxyl functional group on its surface, which resulted in lower interstitial water content and enhanced electrochemical cycling performance. In addition, ball milling synthesis provided the as-prepared Prussian blue with a large surface area, improving its electrochemical rate performance. When used as the cathode of sodium-ion batteries, as-prepared Prussian blue delivered a specific capacity of 145.3 mAh g^−1^ at 0.2 C and 113.7 mAh g^−1^ at 1 C, maintaining 54.5% of the initial capacity after 1000 cycles at 1 C (1 C = 170 mA g^−1^). Furthermore, a solid-state sodium-ion battery was mounted, with as-prepared Prussian blue being employed as the cathode and Na metal as the anode, which delivered a high specific capacity of 128.7 mAh g^−1^ at 0.2 C. The present study put forward an effective solution to overcome the limitations of Prussian blue for its commercial application.

## 1. Introduction

Sodium ion battery are promising for next generation energy storage, which is considered a candidate for the lithium ion battery [1,2,3]. Compared to lithium-ion batteries, sodium-ion batteries have larger ionic diameters and thus more sluggish kinetics. Therefore, it is challenging to design active materials that can be used in sodium-ion batteries, especially cathode materials, because in a full cell, the whole sodium source comes from the cathode. Due to its open framework and suitable ionic/electron pave way, Prussian blue is considered to be a promising candidate for use as a cathode material in sodium-ion batteries and has gained much attention from both academic and industrial communities [4,5,6]. 

Prussian blue is typically synthesized via double iron source co-precipitation, and the influences that anions of ferrous salt have on its structure and performance have been widely investigated. Due to the different reaction dynamics caused by different anions of ferrous salt, different Prussian blue materials exhibit various sizes and shapes, which results in discrepancies in their electrochemical performance [7]. Moreover, the poor electrochemical rate performance of Prussian blue due to the fact that its structure collapses during electrochemical cycling is still a ticklish topic. 

The ball milling synthesis method was utilized to improve its electrochemical rate performance. The irregular particle shape, ultrafine particle size, and large specific surface area of Prussian blue prepared using ball milling can drastically enhance its electron conductivity and improve its electrochemical rate performance [8,9]. Prussian blue can be synthesized using ball milling because the crystal water in the raw materials provides suitable conditions (the synthesis mechanism is similar to that of the co-precipitation process). In the traditional co-precipitation method, the anion radicals in ferrous salts mainly affect the synthesis rate, resulting in different morphologies and particle sizes in the synthesized Prussian blue, thus affecting its electrochemical performance. However, the size and morphology of Prussian blue particles synthesized using ball milling are roughly the same. The influence caused by the anion function group of ferrous salt on Prussian blue prepared by ball-milling has not been investigated yet.

In this study, Prussian blue was synthesized with the ascorbic-acid-assisted ball milling method using three different ferrous salts to determine the influence of the anion functional group. It was revealed that when FeC_4_H_6_O_4_ was used as a raw material, a greater electrochemical performance was displayed in comparison to when other ferrous salts were used. Due to the surface modification caused by the carboxyl functional group, the Prussian blue had lower water content, which further improved its electrochemical cycle performance. This development shows great potential as a low-cost, high-performance, and environmentally friendly technology for large-scale-industrial applications of Prussian blue.

## 2. Results and Discussion

**Figure 1:**
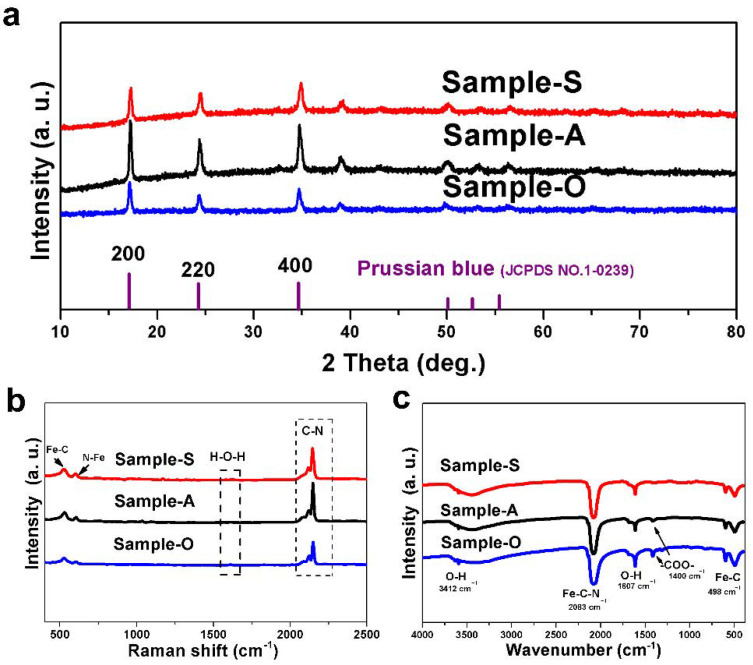
(**a**) XRD patterns of all the samples. (**b**) Raman test of all the samples. (**c**) Fourier transform infrared spectroscopy of all the samples.

The XRD patterns show that all of the as-synthesized samples presented a cubic structure (JCPDS No: 1-0239), as shown in Figure 1a. The XRD pattern of Sample-A delivered a stronger intensity of characteristic peaks than others, indicating higher crystallinity [10,11,12]. The Raman results indicate that all of the samples displayed peaks around 2200 cm^−1^, which were related to the Fe-C-N group, as shown in Figure 1b [13,14]. Figure 1c exhibits all of the samples’ Fourier transform infrared spectra. The peaks that were found at 1605 cm^−1^ and 3412 cm^−1^ were related to H_2_O, and the relative intensity between those peaks and the peaks that related to the Fe-C-N group implies water content in the sample. This result corresponds to the thermo-gravimetric curve presented in Figure S6. The peak observed at 1400 cm^−1^ for Sample-A and Sample-O indicates that the carboxyl functional group attached to the surface of Prussian blue, while the absence of a peak around 1700 cm^−1^ indicates that the whole free-state carboxyl group was washed away during the washing step [15,16,17]. Figure S1a shows the XRD patterns of all of the samples before the washing step, and the result indicates that Prussian blue was mostly synthesized during the ball milling step. Impurities, which consisted of a side reaction product and un-reacted raw materials, were also detected in all of the samples before the washing step. The impurities could be removed from the product during the washing step. Therefore, only Prussian blue was detected in the XRD patterns, which are shown in Figure 1 [8]. Figure S1b,c displays the Fourier transform infrared spectra and Raman spectra of all of the samples before the washing step, which further proves the previously mentioned conclusion. The phenomenon that Sample-A delivers a lower water content than the other two samples may be attributed to the carboxyl functional group modification on the surface of Prussian blue. 

**Figure 2:**
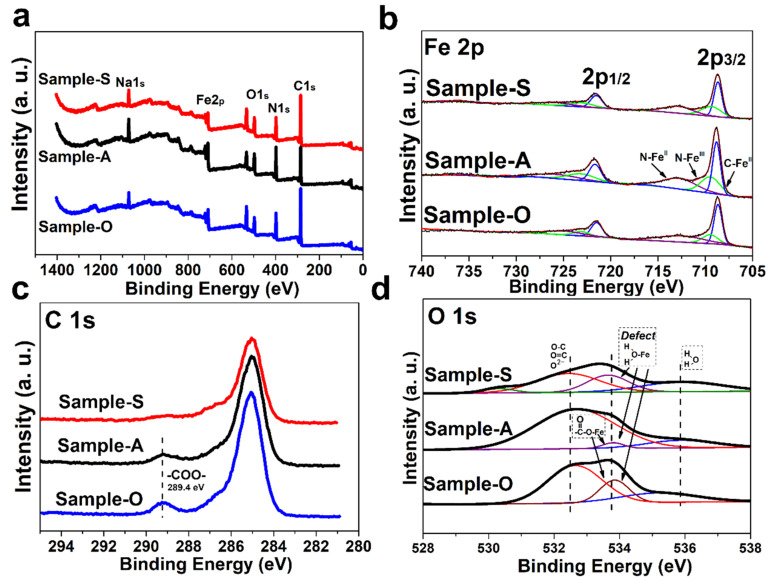
(**a**) XPS wide-scan survey spectrum of all the samples. (**b**) Fe 2p narrow-scan survey spectrum of all the samples. (**c**) C 1s narrow-scan survey spectrum of all the samples. (**d**) O1s narrow-scan survey spectrum of all the samples.

To further prove our hypothesis, the X-ray photoelectron spectroscopy test was performed, and the results are shown in Figure 2 [18]. The XPS wide-scan survey spectrum of all of the samples is presented in Figure 2a, and the narrow-scan survey spectra of specific elements are presented in Figure 2b–d. Figure 2b shows that the combined form iron element was not influenced by the functional group. This indicates that the carboxyl functional group is more likely to chelate with iron elements [19]. The chelation agent function of macromolecule carboxylic acid is widely reported in other literature [20,21]. Figure 2c indicates that the carboxyl functional group could be detected in Sample-A and Sample-O [22]. The characteristic peak of C 1s, related to -OCO-, occurred at about 289 eV (shown in Figure 2c), which corresponded to the FT-IR result [23,24]. The characteristic peak of O 1s, related to the chemical interaction between carboxyl functional groups and Prussian blue (Fe-OCO-), occurred at about 534 eV, which is shown in Figure 2d. This peak is also related to the chemical interaction between interstitial water and Prussian blue (Fe-O-H_2_) [23,24,25]. The characteristic peak of O 1s, related to interstitial water (H-O-H), occurred at about 536 eV [19]. Interstitial water is an unfavorable defect of Prussian blue which deteriorates its electrochemical cycle performance [24]. Figure 2d shows that Sample-A and Sample-O had lower water content due to the chelation of the carboxyl functional group on the surface of the sample (the C-O/C=O/O^2−^ group detected in Sample-S may have been caused by the absorption of oxygen) [23,24,25]. The surface EDS results further prove that, as shown in Figure S2, the content of the iron element is only related to the Fe-C-N-Fe group in Prussian blue. The content of the carbon element is related to the Fe-C-N-Fe group in Prussian blue and the -COO- group in the carboxyl functional group, and the content of the oxygen element is related to interstitial water and the -COO- group in the carboxyl functional group; these details are illustrated in the supporting information. Additionally, at this point, computation was conducted to interpret the chemical mechanism of the decrease in water content, as mentioned in the following section of this article (Table S1). The XPS survey spectrum and EDS results revealed that the carboxyl functional group modification reduced the defect in Prussian blue and lowered the interstitial water content to some extent.

Figures S3 and S4 show the scanning electron microscopy (SEM) images and the transmission electron microscopy (TEM) images for all of the samples. The ball milling process influenced the particle morphology of the Prussian blue obtained; in these samples, nanoscale particles with irregular shapes provided an excellent electrochemical rate performance in comparison to the cube particles, as mentioned in other literature. As shown in other research, the specific surface area also influenced the electrochemical rate performance [26,27]. The adsorption/desorption nitrogen isotherm in Figure S5 and the Barrett Joyner Halenda (BJH) plot in Figure S6 show that the specific surface area of Sample-A reached 91 m^2^ g^−1^, and the average pore size was 3.02 nm. The specific surface area of Sample-O reached 26.5 m^2^ g^−1^, and the average pore size was 3.82 nm. The specific surface area of Sample-S reached 22.4 m^2^ g^−1^, and the average pore size was 3.81 nm. The BET and BJH results show that Sample-A had a larger specific surface area and smaller pore size than the other two samples [28,29]. The larger increase in nitrogen adsorption seen in Sample-A compared to the other two samples may have been due to acetic acid etching [19]. Figure S6d presents the TGA curves for all of the samples, and the results indicate that the samples that underwent carboxyl functional group modification displayed lower water contents [30,31]. This result further proves the conclusion of the XPS results and reveals that the carboxyl functional group can lead to lower interstitial water content in Prussian blue, resulting in a better electrochemical cycle performance. The weight loss of Prussian blue above 300 °C was relate to C-N group decomposition, which exhibited characteristic peaks in the Raman and FT-IR spectra, as shown in Figure 1.

**Figure 3:**
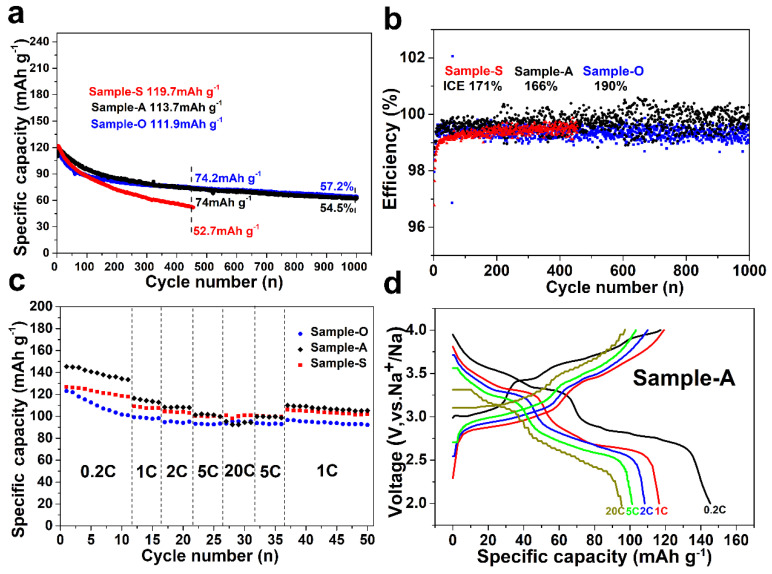
(**a**) Electrochemical cycle performance at 1 C, 1 C = 170 mA g^−1^. (**b**) Efficiency of each electrochemical cycle at 1 C, 1 C = 170 mA g^−1^. (**c**) Electrochemical rate performance from 0.2 C to 20 C, 1 C = 170 mA g^−1^. (**d**) Charge and discharge curves of Sample-A at various current densities.

The electrochemical performance was investigated using half coin cells. Figure 3a displays the electrochemical cycle performance of all of the samples at 1 C current densities (1 C = 170 mA g^−1^). For Sample-A, the electrodes exhibited the specific capacity of 113.7 mAh g^−1^ and maintained 54.5% of the initial capacity after 1000 cycles. For Sample-O, the electrodes exhibited the specific capacity of 111.9 mAh g^−1^ and maintained 57.2% of the initial capacity after 1000 cycles. For Sample-S, the electrode exhibited the specific capacity of 119.7 mAh g^−1^, but it only maintained 44% of the initial capacity after 450 cycles. The improvements in the electrochemical cycle performance are due to the effect of the hydroxyl functional group in Sample-A and Sample-O. The efficiency of per electrochemical cycle is presented in Figure 3b, and the plot of Sample-A was close to one hundred percent. In the full cell test, the efficiency of each electrochemical cycle is an important factor related to the sodium content [32,33]. The initial efficiencies of all the samples were higher than one hundred percent owing to the poor sodium content of obtained Prussian blue, caused by the formation of sodium chloride as the byproducts during the ball milling process. Figure 3c shows the electrochemical rate performance of all of the samples at various current densities from 0.2 to 20 C (specifically at 0.2, 1, 2, 5 and 20 C). Sample-A displayed specific capacities of 145.3, 116.5, 108.3, 101.3, and 95.3 mAh g^−1^, respectively. For Sample-O, the half coin cell delivered specific capacities of 123.1, 99.2, 94.7, 93.1, and 93.5 mAh g^−1^ at 0.2, 1, 2, 5, and 20 C, respectively. For Sample-S, the half coin cell delivered specific capacities of 126.8, 109.2, 104.4, 100.5, and 101 mAh g^−1^ at 0.2, 1, 2, 5, and 20 C, respectively. Figure 3d shows the charge/discharge curves of Sample-A at various current densities. Two relatively flat platforms that were revealed in the curves corresponded to the two obvious redox couples of Fe. The lower platform was attributed to the high-spin Fe bonded with the N atoms of the CN group, and the higher platform was attributed to the low-spin Fe bonded with the C atoms of the CN group. At a current density of 0.2 C, a third platform in the charge/discharge curves of Sample-A was found, which corresponded to the extraction/insertion of Na^+^ from other sites in the structure. This platform gradually disappears in the cycles that followed. This phenomenon was also reported in other literature [32,34]. Figure S8a further shows that a small peak around 3.25 V emerged in the CV curve of Sample-A. Figures S7 and S8 present the charge/discharge curves of the other two samples and their CV curves (the scan ranged from 2.0 V to 4.0 V). Figure S9 shows the electrochemical impedance spectra of all of the samples. The radium of the half circle was related to the charge transfer resistance (Rct), and the intercept of the half circle was related to the series resistance (Rs). The charge transfer resistance of Sample-A was obviously smaller than that of the other two samples. The better electrochemical rate performance of Sample-A was due to the reduced Rct [35,36]. 

**Figure 4:**
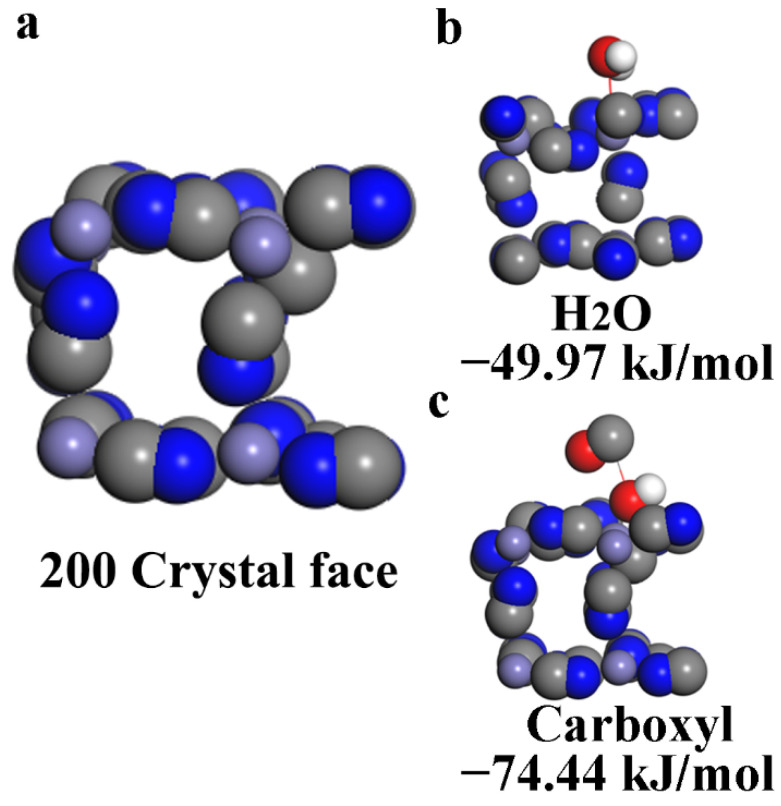
The DFT calculations: (**a**) Prussian blue 200 crystal face. (**b**) Binding energy between interstitial water and Prussian blue. (**c**) Binding energy between carboxyl functional group and Prussian blue.

In the XRD test (Figure 1), the peak related to the (200) facet exhibited the strongest intensity in the Prussian blue obtained. DFT calculations were conducted using the DMol3 program for adsorption on Prussian blue (200) (PB (200)), as shown in Figure 4. It is worth noting that the adsorption energies of H_2_O, COOH, and both adsorbed on the PB (200) facet for obtaining stable structures are calculated, respectively, as shown in Figure 4. The size of the vacuum space was set to be 15 Å to prevent the interaction of atoms. The generalized gradient approximation (GGA) function with a Perdew–Burke–Ernzerh method (PBE) was utilized as the exchange-correlation functional [37]. The double numerical plus polarization (DNP) function was set as the basis set. The effective core potentials (ECP) were used in the calculations. The following convergence criterion were met: 10–5 Ha for total energy, 0.002 Ha/Å for force, and 0.005 Å for displacement. The adsorption energies (Δ E) of molecules on PB (200) were calculated using the following equation: Δ E = Emolecule/PB (200)—Emolecule—EPB (200), where Emolecule/PB (200), Emolecule and EPB (200) are the total energies of the adsorbed molecule on PB (200), the free molecule and bare surface from DFT calculations, respectively. According to this evaluation, the more negative an Δ E value is, the stronger the adsorption capacity of PB (200). This proves that the binding energy between Prussian blue and the carboxyl functional group was stronger than that between water and Prussian blue. Therefore, the modification of the carboxyl group on the surface of Prussian blue would inhibit the formation of interstitial water in the structure.

Furthermore, as solid-state batteries are becoming a hot topic in sodium-ion battery research, a half-cell solid-state battery using Sample A was assembled to test its electrochemical cycling performance (the details are shown in the Experimental Section). The Sample-A solid-state battery delivered a specific capacity of 128.7 mAh g^−1^ in the first cycle and reached a hundred percent initial efficiency at 0.2 C (1 C = 170 mA g^−1^), as shown in Figure S10. For comparison, the electrochemical cycling performance of a Sample-A liquid-state battery was tested at the same current, and it delivered a specific capacity of 145.3 mAh g^−1^ and reached a 135% initial efficiency. The difference in initial efficiency between the solid-state battery and the liquid-state battery may have been caused by the interface problem, which is beyond the scope of this article. The Sample-A solid-state battery delivered the same redox voltage as the Sample-A liquid-state battery, as exhibited in Figure S10b,d. This research reveals that Sample-A has great potential for use in sodium-ion solid-state batteries [38,39]. Table S2 summarizes the differences in the Prussian blue produced in this studies and that in other studies that have been reported in recent years. From this comparison, the specific Prussian blue reveals a great potential in further research [10,32,34,40,41,42,43,44,45,46,47,48].

## 3. Conclusions

Carboxyl-functional-group-modified Prussian blue was synthesized via a simple ball milling method and applied as the cathode in a liquid-state sodium-ion battery. The introduction of a carboxyl functional group was shown to be able to reduce the interstitial water content in Prussian blue to some extent, and it improved the electrochemical cycle performance (for Sample-A, the electrodes exhibited the specific capacity of 113.7 mAh g^−1^ and maintained 54.5% of the initial capacity after 1000 cycles. For Sample-O, the electrodes exhibited the specific capacity of 111.9 mAh g^−1^ and maintained 57.2% of the initial capacity after 1000 cycles, 1 C = 170 mA g^−1^). Sample-A delivered a better electrochemical rate performance (145.3 mAh g^−1^ at 0.2 C and 95.3 mAh g^−1^ at 20 C, 1 C = 170 mA g^−1^) than the other two samples due to its larger surface area and lower charge transfer resistance. Furthermore, Sample-A was shown to have great potential as a candidate material for use in solid-state sodium-ion battery cathodes (128.7 mAh g^−1^ at 0.2 C, 1 C = 170 mA g^−1^). The moderate synthesis method and excellent electrochemical performance mean that Prussian blue has great potential for commercial application.

## 4. Experimental Section

### 4.1. Materials

Ferrous acetate (>90.0% T, Aldrich, Shanghai, China), ferrous oxalate hydrate (99% AR, Aldrich, Shanghai, China), ferrous sulfate (99% AR, Aldrich, Shanghai, China), sodium ferrocyanide (99% AR, Aldrich, Shanghai, China), ascorbic acid (99.5% RT, Aldrich, Shanghai, China), and anhydrous ethanol solution (ACS, ≥99.5%, Aldrich, Shanghai, China) were used. Deionized water was used in the whole synthesis process.

### 4.2. Synthesis of the Cathode Samples

For the synthesis process of Sample-S and Sample-S-NW, in the first step, 4 mmol of FeSO_4_ was heated to remove the crystal water before the ball milling process then milled with 6 mmol of Na_4_Fe(CN)_6_,10H_2_O and 3 mmol of ascorbic acid (the specific powder to raw materials ratio was a 2:1 weight ratio; the rotation speed was set as 250 rpm and operation time was set as 2 h). The obtained mixture was denoted as Sample-S-NW. In the second step, Sample-S-NW was placed into a centrifuge tube to separate the insoluble Prussian blue (the rotation speed was set as 8000 rpm for 3 min) with deionized water (50 mL) and then anhydrous ethanol (50 mL) several times. In the third step, the obtained substance was dried in a vacuum oven at 80 °C overnight (about 8 h). Sample-A and Sample-A-NW were synthesized in the same way as Sample-S and Sample-S-NW, except that Fe(C_2_H_3_O_2_)_2_ was used instead of FeSO_4_ to synthesize Prussian blue in the ball milling process. The synthesis process of Sample-O and Sample-O-NW was the same as that used for Sample-S and Sample-S-NW, except that FeC_2_O_4_ was used instead of FeSO_4_ to synthesize Prussian blue in the ball milling process.

### 4.3. Characterizations of Materials

Raman spectra was obtained using a LabRAM HR800 Evolution with the excitation source of a 532 nm laser. The test spectrum ranged from 400 cm^−1^ to 2500 cm^−1^. Scanning electron microscope imaging (Nova NanoSEM 450, operated at an accelerating voltage of 25 kV.) and transmission electron microscopy (Talos F200X, operated at an accelerating voltage of 200 kV) were conducted to obtain the morphologies and microstructures of all of the samples. FT-IR spectrometry was conducted on VERTEX 70. The test spectrum ranged from 400 to 4000 cm^−1^. The material’s crystal phase was obtained using X-ray diffraction (PANalytical B.V. operated with a Cu Kα radiation). The scan ranged from 10° to 80°, and the scan rate was set at 4 °/min. The thermo-gravimetric analyses (TGA) were carried out Diamond TG, and the temperature ranged from 30 °C to 400 °C at a heating rate of 5 °C min^−1^ in N_2_ atmosphere. XPS spectra were obtained under 200 eV energies, and narrow bands of XPS were obtained under 50 eV using Al Kα radiation (1486.6 eV), mono-chromatized by a couple crystal mono-chromator. An energy-dispersive X-ray spectroscopy (EDS) test was carried out, accompanied by the XPS spectra test under an accelerating voltage of 25 kV. The N_2_ adsorption/desorption isotherms for BET surface area and BJH pore-size distribution were analyzed using a 3H-2000PM2 micro-pore analyzer. The work condition was set under an accelerating voltage of 77K under the N_2_ gas atmosphere.

### 4.4. Electrochemical Measurements

For the cathode electrode, the Prussian blue obtained was used as the active material. Ketjen black and Super P were used as conductive carbon (in order to improve the electrode conductivity). Polyvinylidene fluoride (PVDF) was chosen as the binder. These four materials were mixed in a weight ratio of 7:1:1:1 and then printed on Al foil and dried overnight in a vacuum oven at 80 degrees Celsius. The dried product was cut into many circle plates (0.64 cm^2^) to produce the cathode electrode. The mass load of active material for each electrode was about 1 mg cm^−2^. For the liquid-state sodium-ion battery, the liquid electrolyte consisted of diethyl carbonate (DEC), ethylene carbonate (EC), and fluoroethylene carbonate (FEC) in a volume ratio of 1:1:0.05 with the addition of 1.0 M NaClO_4_. Whatman glass fiber was used as the separator of the liquid-state sodium-ion battery. The assembly process of the liquid-state sodium-ion battery was as follows: Metal Na was chosen as the counter electrode material. The liquid-state battery consisted of a cathode electrode, Ni foam, the liquid electrolyte, the separator, and an anode electrode. The assembly process was performed in a glove box under argon atmosphere (the contents of H_2_O and O_2_ were less than 1 ppm) and sealed under the force of an electrically powered punching machine. After the assembly process, the battery was aged for 6 h before the electrochemical test was carried out. For the solid-state sodium-ion battery, the solid-state electrolyte used Na_3_Zr_2_Si_2_PO_12_ (NZSP) as the basic material. The Na_3_Zr_2_Si_2_PO_12_ (NZSP) was synthesized via a simple ball milling method, using Na_2_CO_3_, NH_4_H_2_PO_4_, SiO_2_, and ZrO_2_ as raw materials and isopropanol as a dispersant. After 12 h of the ball milling process, the product was dried at 70 °C overnight and calcined at 950 °C for 4 h, with the temperature raising rate set at 5 °C min^−1^. After these steps were carried out, the obtained mixture was ball milled for another 7 h (isopropanol was used as a dispersant). Then, it was dried at 70 °C overnight. Finally, the material was cut into a 12 mm diameter circular slice and heated at 1100 °C for 12 h in an air condition in order to sinter the solid-state electrolyte; the raising rate was set at 5 °C min^−1^. The assembly process of the solid-state sodium-ion battery was as follows: Metal Na was chosen as the counter electrode material. The solid-state battery consisted of the cathode electrode, the liquid electrolyte (0.005 mL), the solid-state electrolyte, Ni foam, and the anode electrode. The assembly process was carried out in an argon atmosphere glove box (the contents of H_2_O and O_2_ were less than 1 ppm) and sealed under the force of an electrically powered punching machine. After the assembly process, the battery was aged for 6 h; then, the electrochemical test was carried out.

### 4.5. Electrochemical Characterization

The galvanostatic discharge and charge electrochemical tests (electrochemical cycle performance and electrochemical rate performance) and the Galvanostatic Intermittent Titration Technique (Gitt) test were carried out using a Land CT2001 battery tester (Land Electronics, Landian, Wuhan, China); the test voltage ranged from 2.0 V to 4.0 V. Cyclic voltammetry measurements (CV) were taken on an electrochemical workstation (CHI760E, Chenhua, Shanghai, China) with a frequency range of 0.01–100 Hz and an amplitude of 5 mV. Electrochemical impedance spectroscopy (EIS) was carried out on the electrochemical workstation (CHI760E, Chenhua, Shanghai, China), with high frequency set to 100 KHz and low frequency set to 0.01 Hz with an amplitude of 5 mV.

## Data Availability

Research data are not shared.

## Supplementary Materials

The following supporting information can be downloaded at: https://www.mdpi.com/article/10.3390/nano12081290/s1, Figure S1: Characterization; Figure S2: EDS results of three samples; Figure S3: SEM images of three samples; Figure S4: TEM image of three samples; Figure S5: The adsorption/desorption nitrogen isotherms of three samples; Figure S6: BJH pore size distribution plots and TGA curves; Figure S7: Charge and discharge curve of the Sample-O and the Sample-S at various currents; Figure S8: CV curve of all the samples (range from 2.0 V to 4.0 V); Figure S9: Electrochemical impedance test result; Figure S10: Electrochemical performance; Table S1: Atomic concentrations of each element derived from EDS results; Table S2: Literature Comparison.
