# Supplementary material for: Acid-Assisted Ball Mill Synthesis of Carboxyl-Functional-Group-Modified Prussian Blue as Sodium-Ion Battery Cathode
Source: Nanomaterials (Basel). 2022 Apr 11;12(8):1290. doi: 10.3390/nano12081290 (PMC9025874; doi:10.3390/nano12081290)
Supplement: Supplementary file 1 [file nanomaterials-12-01290-s001.zip › nanomaterials-1664862-supplementary.pdf]

nanomaterials

Supplementary Materials

Acid-Assisted Ball Mill Synthesis of Carboxyl-Functional-Group-Modified Prussian Blue as Sodium-Ion Battery Cathode

Yu Luo, Jiayu Peng, Shengming Yin *, Lihong Xue * and Youwei Yan *

State Key Laboratory of Material Processing and Die & Mould Technology, School of Materials Science and Engineering, Huazhong University of Science and Technology, Wuhan, 430074, China; luoyu@hust.edu.cn (Y.L.); d201980327@hust.edu.cn (J.P.)

* Correspondence: yinsm@hust.edu.cn (S.Y.); xuelh@hust.edu.cn (L.X.); yanyw@hust.edu.cn (Y.Y.)

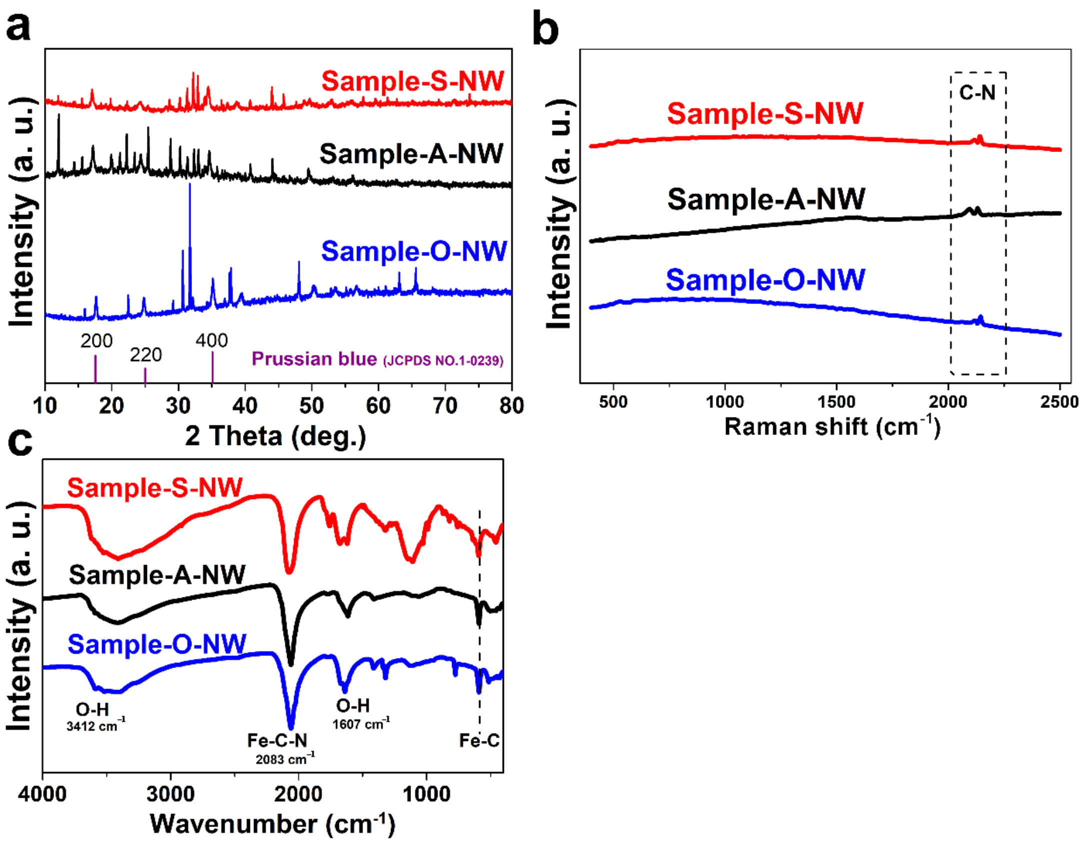

Figure S1. Characterization: (**a**)XRD pattern. (**b**)Raman spectrum result. (**c**) Fourier transform infrared spectroscopy result.

Sample-S

Quantify By: Components

Component	BE [eV]	FWHM [eV]	RSF	Atomic conc. [%]	Error [%]	Mass conc. [%]	Error [%]
Fe 2p	708.79	0.00	2.96	8.1	0.50	26.4	1.29
O 1s	533.04	0.00	0.78	17.6	0.65	16.3	0.64
C 1s	285.04	0.00	0.28	65.6	0.79	45.8	1.02

Sample-A

Quantify By: Components

Component	BE [eV]	FWHM [eV]	RSF	Atomic conc. [%]	Error [%]	Mass conc. [%]	Error [%]
Fe 2p	708.64	0.00	2.96	4.2	0.42	15.5	1.37
O 1s	532.69	0.00	0.78	18.6	0.54	19.6	0.63
C 1s	285.04	0.00	0.28	72.0	0.65	57.0	1.09

Sample-O

Quantify By: Components

Component	BE [eV]	FWHM [eV]	RSF	Atomic conc. [%]	Error [%]	Mass conc. [%]	Error [%]
O 1s	533.66	0.00	0.78	16.4	0.50	17.3	0.56
C 1s	285.06	0.00	0.28	74.7	0.59	59.1	0.99
Fe 2p	708.71	0.00	2.96	4.6	0.37	17.1	1.19

Figure S2. EDS results of three samples

Table S1. Atomic concentrations of each element derived from EDS results.

	Fe (atomic conc)	O (atomic conc)	C (atomic conc)	Fe/C (factor x)	O/C (factor y)
**Sample-S**	**8.1**	**17.6**	**65.5**	**0.124**	**0.268**
**Sample-A**	**4.2**	**18.6**	**72**	**0.058**	**0.258**
**Sample-O**	**4.6**	**16.4**	**74.7**	**0.062**	**0.22**

Factor x set as the ratio Fe/C, as Fe atom content come from Prussian blue, C atom content come from Prussian blue and carboxyl function group, the decrease of the factor x exhibit that more C atom on the surface of the Prussian blue.

Factor y set as the ratio O/C, as C atom content come from Prussian blue and carboxyl function group, O atom content come from carboxyl function group and interstitial water.

We set Prussian blue content to 1 mol, and set carboxyl function group content to α mol, set interstitial water content (the defect) to β mol.

As the carboxyl function group (O element content/C element content = 2/1), get the formula: $y = (\beta + 2\alpha)/(1 + \alpha)$

then, $\beta = y + (y - 2)\alpha$,

as $y < 1$, $\alpha \geq 0$, we get the conclusion that $\beta_{\text{sample-O}} < \beta_{\text{sample-A}} < \beta_{\text{sample-S}}$.

The result reveal that the decrease of the factor y mean less interstitial water on the surface of the Prussian blue

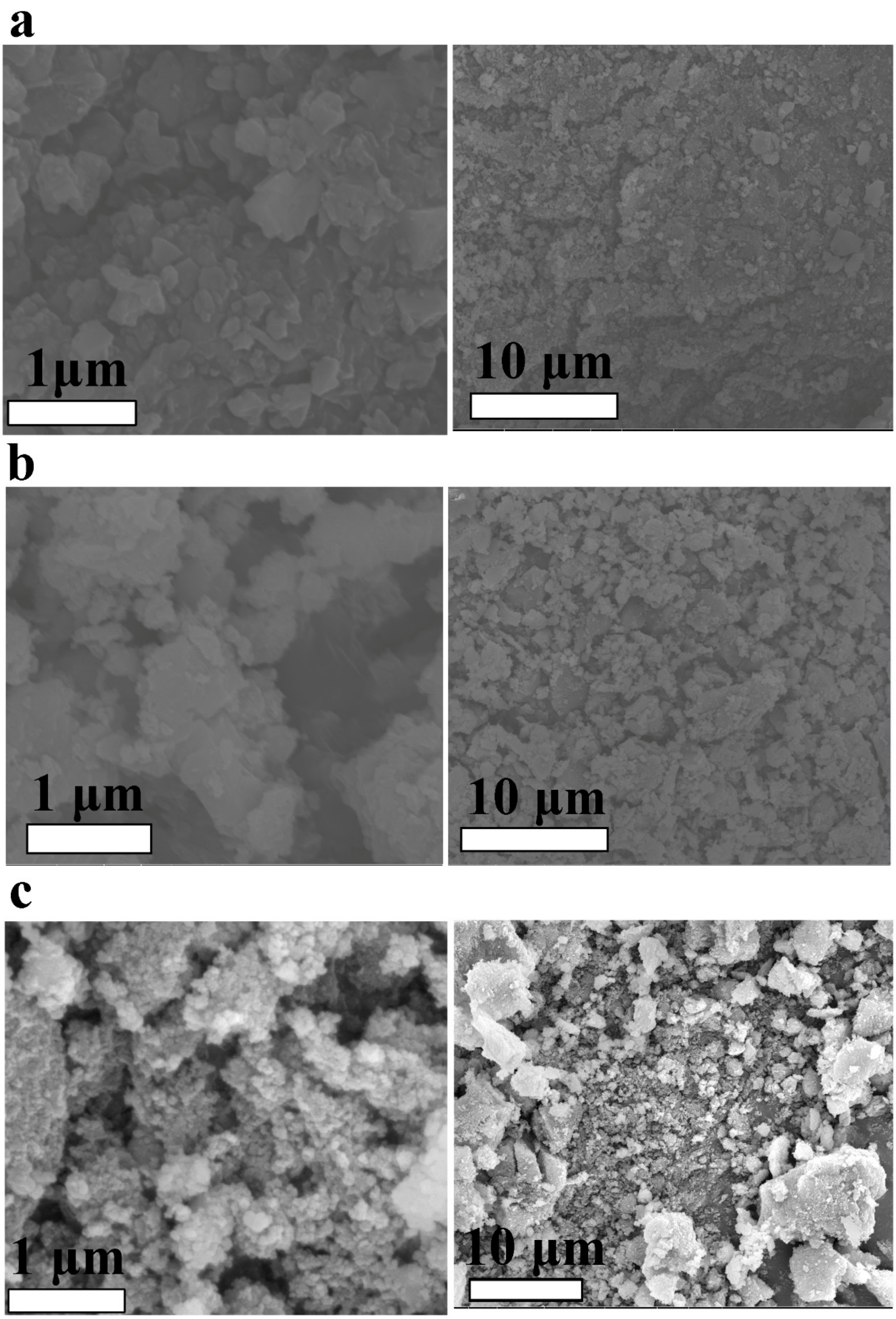

Figure S3. SEM images of three samples. (**a**) The Sample-S. (**b**) The Sample-A. (**c**) The Sample-O.

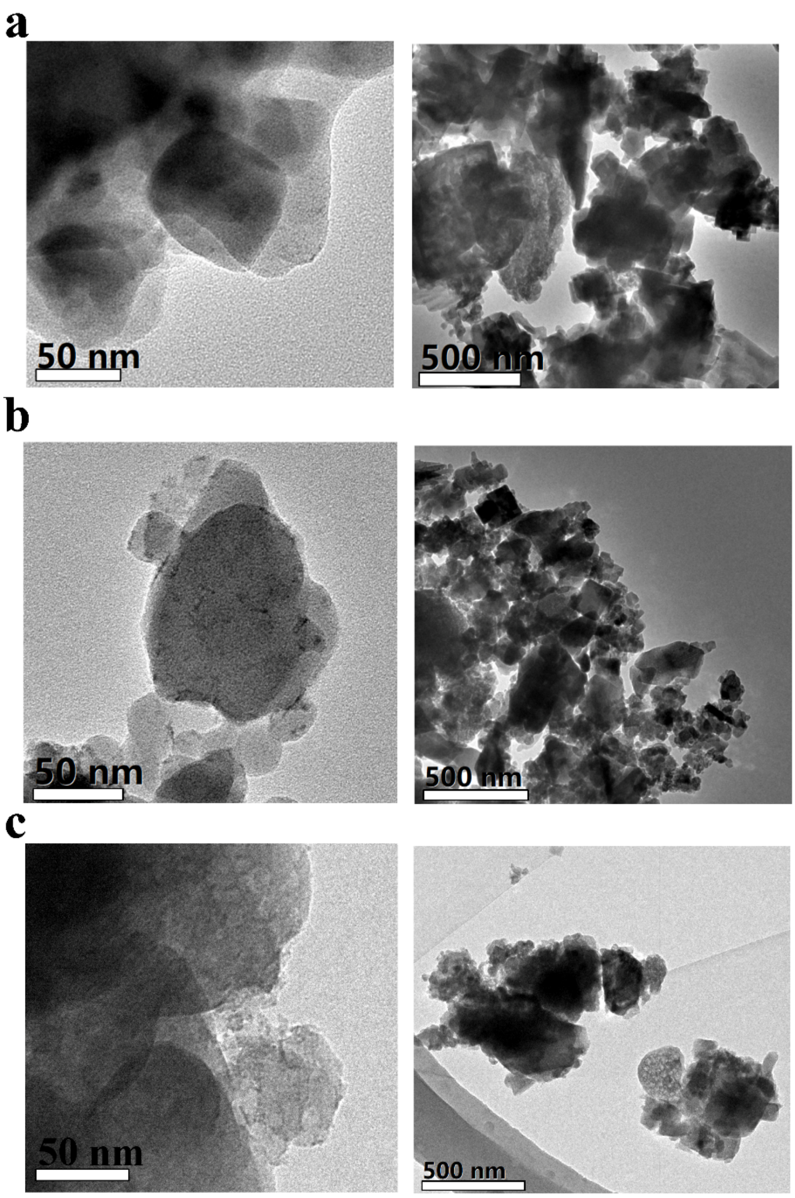

Figure S4. TEM image of three samples. (**a**) The Sample-S. (**b**) The Sample-A. (**c**) The Sample-O.

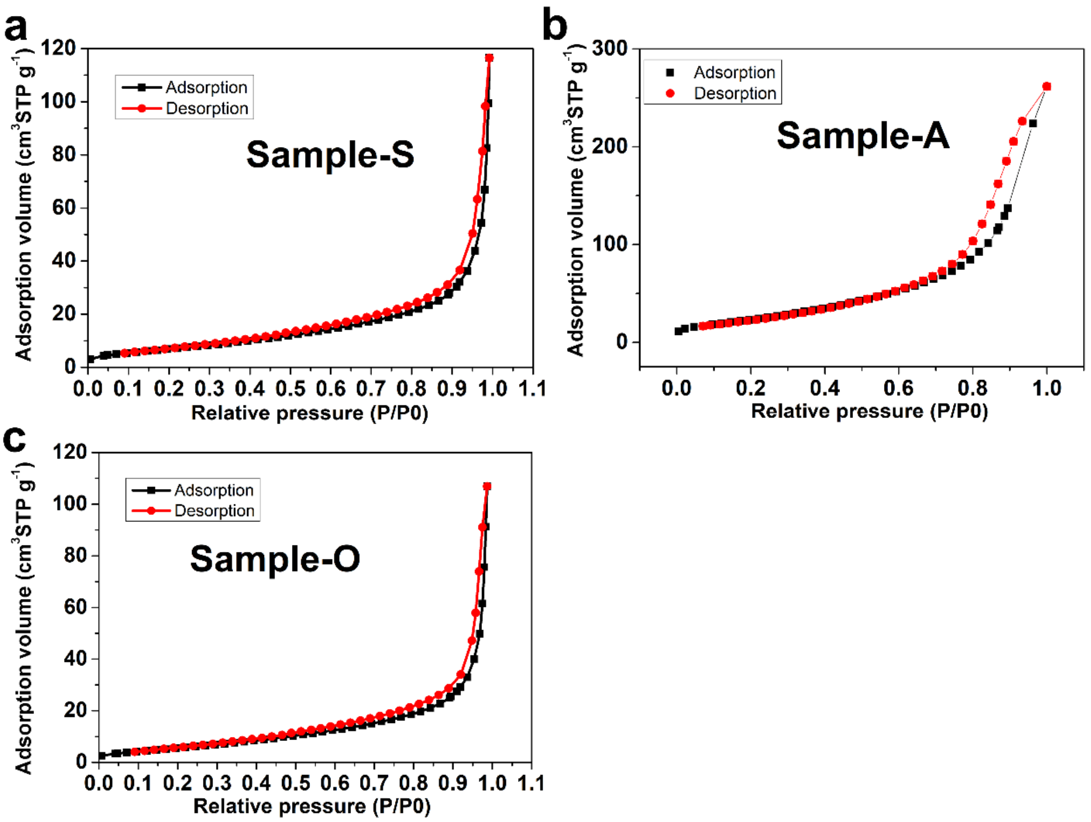

Figure S5. The adsorption/desorption nitrogen isotherms of three samples. (**a**) The Sample-S. (**b**) The Sample-A. (**c**) The Sample-O.

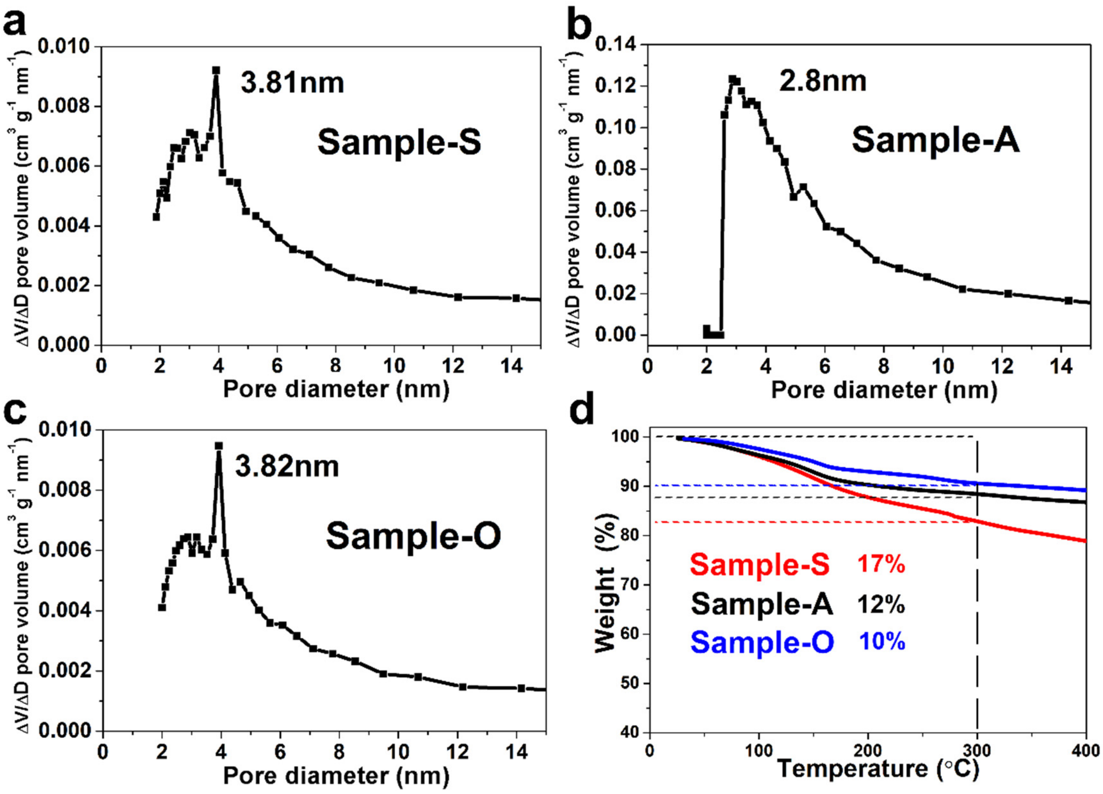

Figure S6. BJH pore size distribution plots and TGA curves. (**a**) The Sample-S. (**b**) The Sample-A. (**c**) The Sample-O. (**d**) Temperature.

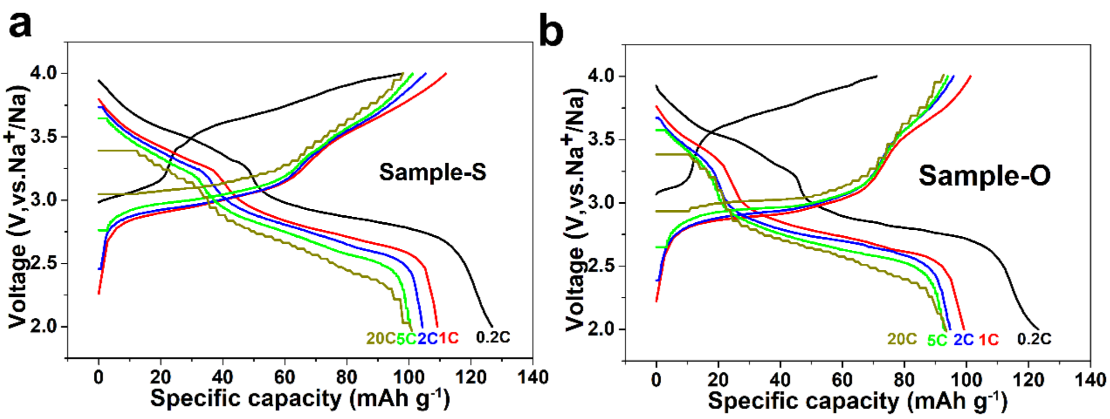

Figure S7. Charge and discharge curve of the Sample-O (**a**) and the Sample-S (**b**) at various currents.

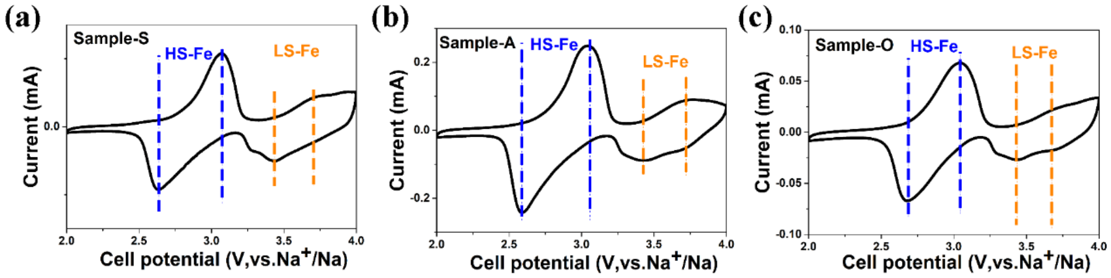

Figure S8. CV curve of all the samples (range from 2.0V to 4.0V). .(**a**) The Sample-S. (**b**) The Sample-A. (**c**) The Sample-O.

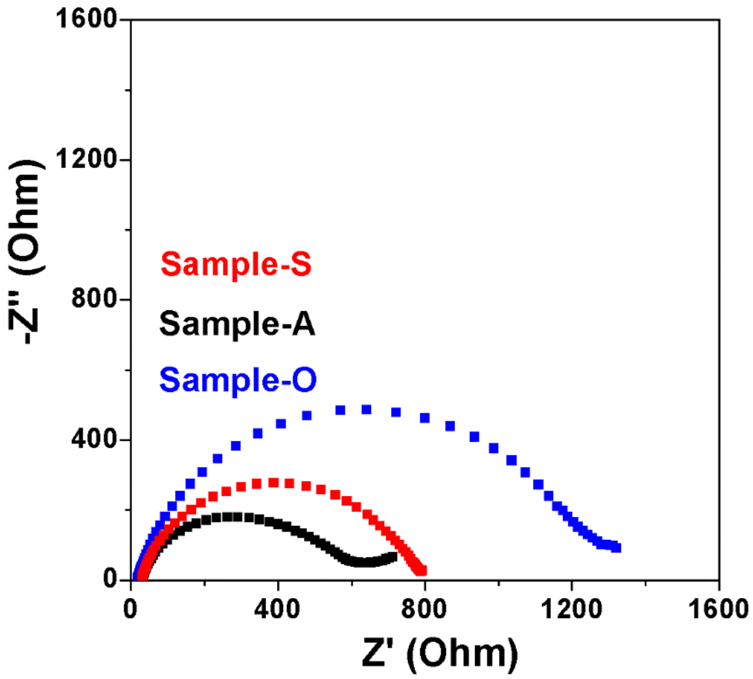

Figure S9. Electrochemical impedance test result.

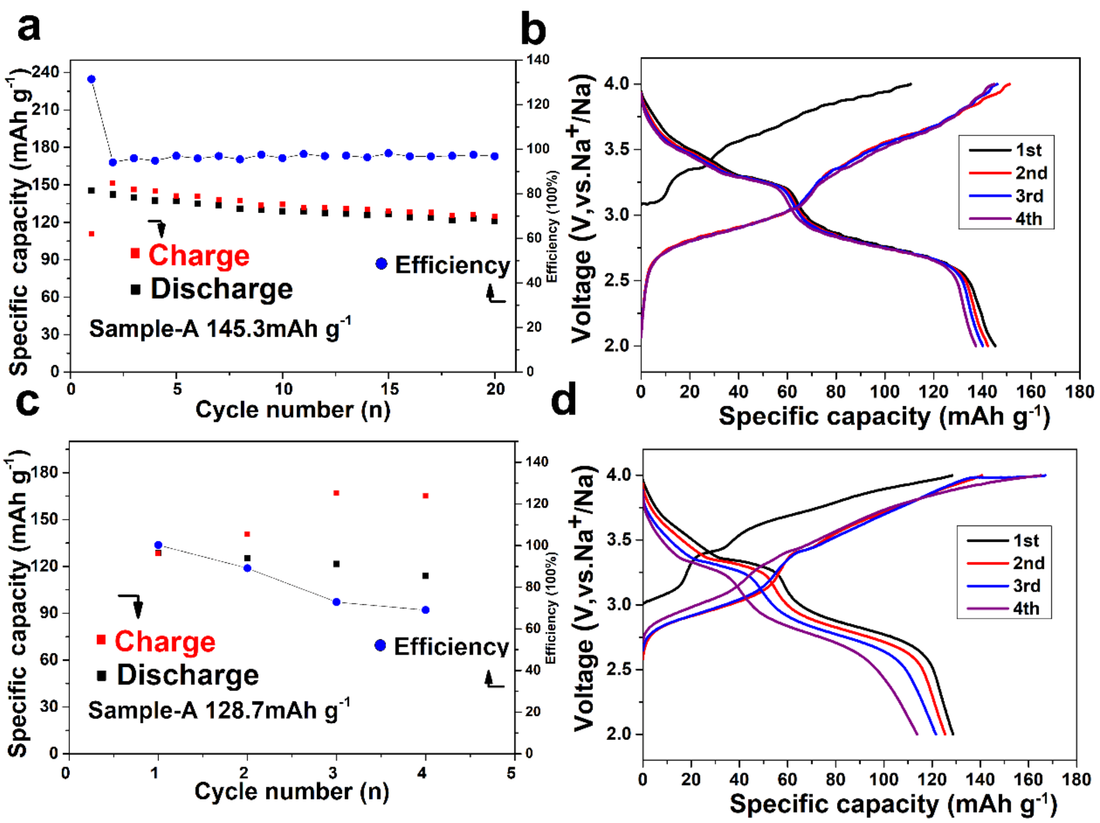

Figure S10. Electrochemical performance: (**a**) and (**b**) Liquid state half cell electrochemical performance at 0.2 C, 1 C=170 mA g^{-1}. (**c**) and (**d**) Solid state half cell electrochemical performance at 0.2 C, 1 C=170 mA g^{-1}.

Table S2. Literature Comparison.

Synthesis method	Synthetic hazard level	Raw materials	Additive	Liquid state sodium ion battery		Solid state sodium ion battery	Year	[Ref.]
				cycle performance current@cycle @retention	rate performance	electrochemical performance		
**Co-precipitation**	**Moderate**	**Double Fe-ion source:FeSO$_4$.7 H$_2$O and Na$_4$Fe(CN)$_6$•10 H$_2$O**	**Sodium citrate, NaCl**	**5C@3700@79%**	140mAh/g at 0.2C,130mAh/g at 1C, 105mAh/g at 5C	/	2020	[40]
Hydrothermal reaction	Danger (H$_2$SO$_4$ made Prussian blue produce HCN)	Single Fe-ion source: Na$_4$Fe(CN)$_6$•10 H$_2$O	Polyvinylpyrrolidone, H$_2$SO$_4$	800mA/g@4.00 @80%	113mAh/g at 1600mA/g, 139mAh/g at 50 mA/g	/	2020	[41]
Co-precipitation	Moderate	Double Fe-ion source: Na$_4$Fe(CN)$_6$•10 H$_2$O and MnSO$_4$•H$_2$O	NaCl	1C@500@91.8 %	142.7mAh/g at 0.1C, 89.7mAh/gat 20C	/	2018	[42]

Co-precipitation	Danger (HCl made Prussian blue produce HCN)	Single Fe-ion source: $Na_4Fe(CN)_6$•10 H_2O	HCl	0.05A/g@100@84.5%	51.1 mAh/g at 0.4 A/g 49.7 mAh/g at 1 A/g	/	2014	[43]
Co-precipitation	Danger	Single Fe-ion source: $Na_4Fe(CN)_6$•10 H_2O	PVP, Ascorbic acid, Sodium dodecyl benzene sulfonate, Acetone and Acetic acid	100mA/g@100@70%	103 mAh/g at 25 mA/g 80 mAh/g at 400 mA/g	/	2019	[34]
Co-precipitation	Danger (HCl made Prussian blue produce HCN)	Single Fe-ion source: $Na_4Fe(CN)_6$•10 H_2O	/	25mA/g@150@97%	160mAh/g at 25mA/g 110mAh/g at 150 mA/g 70mAh/g at 600mA/g	/	2014	[44]
Co-precipitation	Moderate	Double Fe-ion source: $FeSO_4.7H_2O$ and $Na_4Fe(CN)_6$•10 H_2O	Sodium citrate	100mA/g@500@71%	116mAh/g at 10mA/g 70mAh/g at 2000mA/g	/	2020	[32]
Co-precipitation	Danger (HCl made Prussian blue produce HCN)	Single Fe-ion source: $Na_4Fe(CN)_6$•10 H_2O	HCl, NaCl	20mA/g@400@97%	103.6mAh/g at 20mA/g, 90mAh/g at 100mA/g	/	2015	[46]
Co-precipitation	Danger (HCl made Prussian blue produce HCN)	Single Fe-ion source: $Na_4Fe(CN)_6$•10 H_2O	HCl, Ascorbic acid	25mA/g@200@90%	150mAh/g at 25mA/g	/	2015	[10]
Co-precipitation	Moderate	Double Fe-ion source: $FeCl_2$•4 H_2O and $Na_4Fe(CN)_6$•10 H_2O	/	200mA/g@100@70.5%	129mAh/g at 200mA/g, 71.2mAh/g at 1200mA/g	/	2015	[47]
Co-precipitation	Moderate	Double Fe-ion source: $FeCl_2$•4 H_2O and $Na_4Fe(CN)_6$•10 H_2O	NaCl	1C@200@73%	123 mAh/g at 100mA/g,78mAh/g at 8C	/	2016	[48]
Ball mill	Moderate	Double Fe-ion source: Ferrous acetate and	Ascorbic acid	1C@450@65%	145.3mAh/g at 0.2C 113.3mAh/g at 1C	Fe-Fe PB: 128.7mAh/g at 0.2C	This work	/

		$Na_4Fe(CN)_6$•10 H_2O			95.3mAh/g at 20C (1C=170mA/g)			
Co-precipitation	Moderate	Double ion source: $Ni(CH_3COO)_2$ and $Na_4Fe(CN)_6$•10 H_2O	Sodium citrate	0.1A/g@1120@ 85.5%	73.8mAh/g at 0.05A/g 54.5mAh/g at 0.5A/g	Ni-Fe PB: 73.8mAh/g at 0.05A/g	2020	[35]
Co-precipitation	Danger (HCl made Prussian blue produce HCN)	Single Fe-ion source: $Na_4Fe(CN)_6$•10 H_2O	HCl	/	70 mAh/g at 6C	Fe-Fe PB: 87.5mAh/g at 8C	2020	[45]
